# Unveiling Novel Traits Associated with Ulcerative Colitis via Phenome-Wide Associations Enhanced by Polygenic Risk Statistics

**DOI:** 10.3390/genes16121431

**Published:** 2025-11-30

**Authors:** Yiming Wu, Ling Liu, Meltem Ece Kars, Rui Li, Menglong Li, Yuval Itan

**Affiliations:** 1 College of Life Science, China West Normal University, Nanchong 637009, China; lirui041112@gmail.com; 2 Key Laboratory of Southwest China Wildlife Resources Conservation (Ministry of Education), China West Normal University, Nanchong 637009, China; 3 College of Chemistry, Sichuan University, Chengdu 610065, China; liuling199801@163.com (L.L.); liml@scu.edu.cn (M.L.); 4 The Charles Bronfman Institute for Personalized Medicine, Icahn School of Medicine at Mount Sinai, New York, NY 10029, USA; meltemece.kars@mssm.edu; 5 Department of Genetics and Genomic Sciences, Icahn School of Medicine at Mount Sinai, New York, NY 10029, USA

**Keywords:** ulcerative colitis, association analysis, risk prediction, PRS-PheWAS

## Abstract

**Background**: Ulcerative colitis (UC) is a major form of inflammatory bowel disease affecting the gastrointestinal tract. Increasing evidence suggests UC is predisposed to co-occurring with other autoimmune diseases, yet its pathogenesis remains insufficiently understood. Large-scale biobank-based cross-trait genetic analyses may provide insights into the origins of UC and its comorbidities. **Methods**: Using the UK Biobank and Mount Sinai BioMe Biobank, we conducted genome-wide association studies (GWASs) in individuals of European ancestry. High-impact rare variants were aggregated for collapsing analysis. Genome-wide significant variants were tested in a phenome-wide association study (PheWAS) to explore UC comorbidities. Polygenic risk scores (PRSs) were derived from large-scale GWASs under different thresholds and functionalities, and the best-performing PRS was further applied in a PRS-based PheWAS. Genetic correlation between UC and highly associated traits was evaluated. **Results**: GWASs identified four genome-wide significant loci, including two novel variants (rs2314757, *p* = 4.82 × 10^−11^, OR = 0.81; rs6869382, *p* = 2.48 × 10^−8^, OR = 0.83) and two previously reported UC-associated sites (rs4654925, *p* = 1.85 × 10^−8^, OR = 0.84; rs2836882, *p* = 1.23 × 10^−11^, OR = 0.78) outside the HLA region. The optimal PRS, constructed with SNPs at *p* < 0.05, conferred an odds ratio of 5.86 (95% CI: 5.05–6.86) for UC in individuals with the highest versus lowest quintile. Both variant- and PRS-based PheWASs consistently highlighted type 1 diabetes (T1D) as the most significant comorbidity, confirmed by genetic correlation analysis. **Conclusions**: This study reveals novel loci contributing to UC and highlights comorbidities with shared genetic bases. UC PRSs demonstrated strong utility beyond risk prediction, effectively identifying UC-associated traits. A robust genetic correlation was established between UC and T1D.

## 1. Introduction

Ulcerative colitis (UC), one of the major forms of inflammatory bowel disease (IBD), is a chronic idiopathic intestinal disorder that significantly impairs patients’ quality of life [1,2]. UC shows the highest reported prevalence in Europe, and its incidence continues to rise worldwide [3]. Although the etiology of UC remains incompletely understood, genetic risk factors are considered to play a major role in its development [1]. Genome-wide association studies (GWASs) have identified multiple genomic loci associated with UC, thereby advancing our understanding of its genetic architecture and etiology [4,5].

UC is classified as an autoimmune disease, and several shared genetic risk factors have been observed across autoimmune conditions [6,7]. Shared genetic architecture can lead to shared traits, overlapping disorders, or comorbidities, defined as the co-occurrence of two diseases in the same individual. Consequently, patients with IBD often experience additional unrelated diseases, which increase disease burden and complicate diagnosis, treatment, and disease management [8]. UC patients are reported to have higher rates of multimorbidity compared with those with Crohn’s disease [9,10]. Therefore, early diagnosis, stratification, and intervention are critical for UC therapies and medical management. Leveraging genetic data to identify high-risk individuals is one approach to early stratification. In this context, polygenic risk scores (PRSs) can be used to predict patient outcomes by summarizing the cumulative effect of risk alleles derived from large-scale GWASs across different populations [11].

As a computationally derived biomarker integrating diverse genetic variants, a PRS has substantially greater predictive power than individual single nucleotide polymorphisms (SNPs). When combined with phenome-wide association studies (PheWASs), a PRS can further extend its utility. PheWASs provide a valuable framework for simultaneously investigating genetic variants alongside physiological and clinical phenotypes, thereby enabling the exploration of associations across a wide range of traits. This framework can be extended to assessing relationships between multiple phenotypes and the genetic liability to a given trait by applying a PRS-PheWAS approach. To date, no studies have examined the correlations between the genetic predisposition to UC and related phenotypes using PRS-PheWASs.

In the present study, we investigate genetic factors contributing to UC risk by integrating genetic data and electronic health records from two large-scale biobanks. We applied both single-variant and collapsing approaches to identify the genetic liability to UC. PRSs were generated using SNP sets from large-scale GWASs, as well as functionally annotated SNPs. The best-performing PRS was subsequently applied to identify UC-associated traits through a PRS-PheWAS. Finally, causal relationships between UC and correlated traits were assessed using genetic correlation and causal inference analyses.

## 2. Materials and Methods

### 2.1. Sample Collections

**Figure 1:**
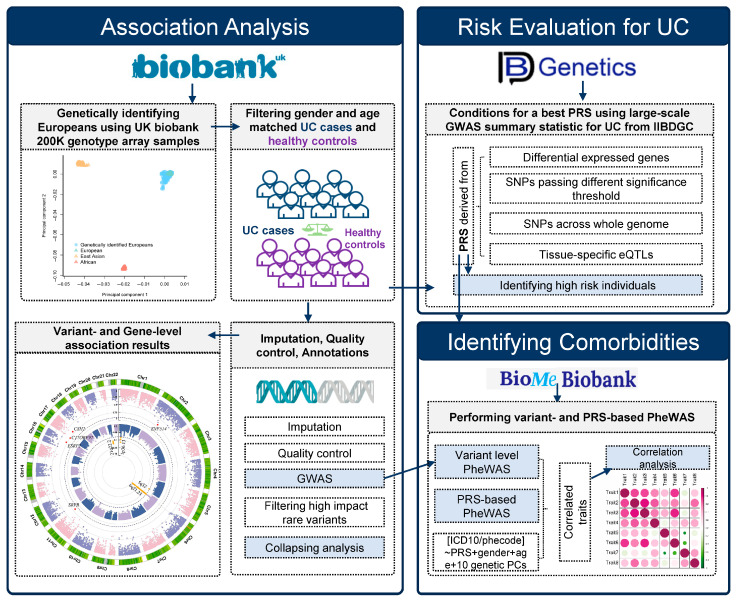
Workflow of the current study. Individuals of European ancestry were genetically identified from the UK Biobank. Following imputation using a European reference panel and quality control, gender- and age-matched cases and controls were defined based on ICD-10 codes for both association analysis and polygenic risk score (PRS) evaluation. Common variants and selected high-impact rare variants were analyzed using genome-wide association study and variant collapsing analysis, respectively. Subsequently, lead SNPs and tag genes associated with ulcerative colitis (UC) were identified through gene function proximity analysis. The most significantly associated pathway was then determined using Ingenuity Pathway Analysis (IPA, version 01-23-01) on the resulting gene set. To develop a discriminative PRS, we applied multiple filtering conditions to SNPs from a large-scale UC genome-wide association study (GWAS), conducted by the International Inflammatory Bowel Disease Genetics Consortium (IIBDGC). The PRS was then evaluated in our UC cohort. Using the Mount Sinai BioMe Biobank, we further utilized significant variants and PRS results from our UC cohort to conduct variant-based and PRS-based phenome-wide association studies (PheWASs), aiming to identify potential comorbidities of UC. Finally, we assessed genetic correlations between UC and co-occurring conditions with UC identified from PheWASs.

The UC cases were identified based on the diagnostic information from the UK Biobank [12]. Individuals diagnosed with ICD10 code starting with “K51” were deemed UC cases. We obtained 4350 UC samples from 500 K genotype array samples, of which 2260 (51.95% of the cases) were males. We constructed an age- and gender-matched control group by selecting 4350 non-IBD samples. After implementations of QC procedures and the identification of Europeans (Figure S1), 3999 cases and 3996 controls were retained for downstream analysis (Figure 1). No significant deviations were observed between cases and controls in terms of age (*p* = 0.72 in Student’s two-sided *t*-Test) and gender (*p* = 0.798, Pearson’s Chi-squared test). The type 1 diabetes cases were collected by ICD-10 code E10, and gender- and age-matched controls were filtered from non-IBD and non-T1D individuals. We eventually obtained 1303 cases and 1301 controls after the quality control process.

### 2.2. Quality Control

Quality control (QC) procedure was performed on the original genotype array data. Variant-level and sample-level filtering were performed using PLINK (V1.9) in the following steps [13]: (1) removing individuals with sex inconsistency between self-reported and genotype-measured; (2) excluding individuals with a call rate < 94%; (3) removing individuals with an inbreeding coefficient exceeding ± 6 SD of the mean inbreeding coefficients; (4) for each pair of duplicated or related individuals (first- and second-degree relatives, PI_HAT > 0.25), the subject with the higher missingness rate was removed.; (5) identifying Europeans from cases and controls and excluding non-European samples; (6) excluding SNPs on the X, Y, and mitochondrial chromosomes; (7) removing variants with a call rate below 95%; (8) removing monomorphic variants; (9) removing variants with missing rate differences exceeding 0.05 between case and controls; (10) removing variants in violation of Hardy–Weinberg equilibrium with a *p*  <  10^−6^ in controls; and (11) flipping the SNPs on the reverse strand.

### 2.3. Genotype Imputation

Imputation was performed based on the genotyped SNPs that passed the QC criteria utilizing the Michigan Imputation Server (Minimac4) [14].The Beagle 5.4 was employed in order to estimate the haplotypes, while the Haplotype Reference Consortium r1.1 was used as the reference panel. SNPs with an imputation quality score (r^2^) value below 0.8 were excluded. Only SNPs that passed QC were retained for the imputation process. Afterward, we removed SNPs in violation of Hardy–Weinberg equilibrium with a *p*  <  10^−6^ in controls again.

### 2.4. Variant Annotation

All variants were annotated using Variant Effect Predictor (VEP, v109) run locally [15]. The CADD (v1.6) plugin was employed for further assessing the pathogenicity of variants [16]. All annotation processes were conducted based on the NCBI Genome Reference Consortium Human Build 37 (GRCh37) genome coordinates, consistent with the genome build used in native UK Biobank imputation. The most severe category was selected for variants with multiple annotation results from alternative gene isoforms.

### 2.5. Genome-Wide Association Study

We conducted an association analysis at the variant level, focusing on variants with a MAF ≥ 0.01. The variant-level association analysis was performed using a logistic regression model in PLINK (V1.9). Age, biological sex, and the first 10 PCs derived from principal component analysis (PCA) on all cases and controls were included as covariates in the association analysis [17]. Linkage disequilibrium-based (LD-based) clumping was calculated using PLINK (V1.9), with a significance threshold for the index SNP (clump-p1) of 0.00001 and a secondary significance for clumped SNPs (clump-p2) of 0.001 in a range of 150 kilo base pairs (kbp).

### 2.6. Variant Collapsing Analysis

The rare variants (MAF < 0.01) with high impact were retained for variant collapsing analysis. High-impact variants were selected according to the “consequence” field of VEP results. The high-impact variants consist of 14 types of mutations: “missense variant”, “start lost”, “stop lost”, “stop gained”, “splice_acceptor_variant”, “splice_donor_variant”, “inframe_insertion”, “inframe_deletion”, “protein_altering_variant”, “start_retained_variant”, “stop_retained_variant”, “stop_gained”, “stop_lost”, “regulatory_region_variant”, and “frameshift_variant”. Variants located in low-complexity regions (LCRs) were filtered out. LCRs were identified using the RepeatMasker track of UCSC table browser (https://genome.ucsc.edu/cgi-bin/hgTables, accessed on 26 March 2025). We applied a MAF of 0.01 to filter rare variants using gnomAD European allele frequency as a reference. The optimized method of Sequence Kernel Association Test (SKAT-O) was employed for running variant collapsing analysis.

### 2.7. Pathway Analysis and Biological Distance Evaluation

For 155 significant genes (*p* < 0.01) resulting from variant collapsing analysis, we conducted pathway analysis using Ingenuity Pathways Analysis (IPA, version 01-23-01) [18]. To further prioritize the candidate genes, human gene connectome (HGC, online version at https://hgidsoft.rockefeller.edu/HGC/) [19] was employed to calculate the biological distance between each candidate gene to 341 UC-associated genes collected from the GWAS catalog [20] database under EFO ID of EFO_0000729. The average distances between the candidate gene to all UC genes were utilized to prioritize the candidate genes.

### 2.8. Phenome-Wide Association Analysis

To investigate the potential pleiotropy of UC and other diseases in the EHR phenome, we performed different PheWAS calculations, so-called variant-level- and PRS-based- PheWASs, using the Mount Sinai BioMe BioBank. The analysis was performed on 2309 mapped representative EHR phenotypes, each with a minimum of 50 cases, from 53,449 participants after quality control (QC). The case group for a given ICD10 code was defined by the presence of at least 1 assignment of the ICD code from the EHR. Controls for each ICD10 were defined by the absence of the same ICD codes that defined cases and the presence of at least 1 assignment of the ICD code Z00.00 (encounter for general adult medical examination without abnormal findings). The control group size was set to approximately five times the number of cases for each phenotype after removing overlapping samples, to prevent effect size estimation bias due to data imbalance. Firstly, we performed a variant-level PheWAS for the 25 tag SNPs in clumps after clumping the genome-wide significant variants (*p* = 5 × 10^−8^). Additionally, to estimate the contrasting performance of polygenic prediction over the single-variant approaches, we performed a PRS-based PheWAS utilizing the variants derived from the most optimal PRS model [21]. The association between the UC PRS and clinical phenotypes was assessed by logistic regression with the following model: logit(Clinical Phenotype = 1|PRS, gender, age, 10 PCs) = b0 + b1 × PRS + b2 × Gender + b3 × Age + b4 × 10 PCs. This analysis aimed to compare phenotypes associated with the cumulative effects captured by PRSs with those linked to individual genetic signals identified in the genome-wide association analysis. In this study, phenome-wide significance was defined as a stringent Bonferroni-corrected threshold of *p* = 1.43 × 10^–5^ adjusting for multiple testing, calculated by dividing *p* = 0.05 by the 3496 phenotypes interrogated. In order to obtain more clinically meaningful PheWAS results, we also performed variant- and PRS-based PheWAS using phecodes. ICD-10 codes were grouped into relevant phecodes according to Phecode map version 1.2. This grouping resulted in 1856 phecodes, yielding a phenome-wide significance of 2.69 × 10^−5^. All PheWAS analyses were adjusted for age, gender, and the first 10 PCs of the PCA results. Manhattan PheWAS plots of -log10 (*p*-value) were generated using the “CMplot” and “ggplot” packages in the R (V4.1.3) environment.

### 2.9. Polygenic Risk Score Based on Different SNP Sets

SNPs result from large-scale GWASs. We obtained the full summary statistics from the latest large meta-GWAS on IBD through the International Inflammatory Bowel Disease Genetics Consortium (IIBDGC, https://www.ibdgenetics.org, accessed on 5 May 2025) in order to construct a PRS for UC. The meta-GWAS was conducted on individuals of East Asian and European ancestries, which included 16,390 UC cases and 338,106 controls of Europeans [4]. For PRS generation, in addition to the standard QC, we retained only biallelic variants with a MAF ≥ 0.01 that were present in both our genotype data and the GWAS summary statistics. The positions of reported variants are based on GRCh37, consistent with the reference genome utilized in UK Biobank imputation. All 56 significant markers identified in the GWAS meta-analysis were used for generating a PRS; however, 9 SNPs were not present in our genotype data; therefore, scores were constructed using the remaining 47 markers. In addition, we used genome-wide SNPs and employed the “clumping + thresholding” method to generate PRSs for individuals using PLINK (V1.9). To compare the predictive accuracy across different sets of SNPs, we applied the clumping and thresholding method with varying parameters. By setting the clumping *p*-value to 1, we tested multiple LD r^2^ thresholds (0.05, 0.1, 0.2, 0.4, 0.6, 0.8) for assigning SNPs within a 250 kb window to clumps. Additonally, we used nine *p*-value cutoffs (5 × 10^−8^, 5 × 10^−7^, 5 × 10^−6^, 5 × 10^−5^, 5 × 10^−4^, 5 × 10^−3^, 5 × 10^−2^, 0.5, 1) to SNP inclusion.

SNP in differentially expressed genes. Genes meeting the criterion of |log2FoldChange| > 1 and adj. *p*-value < 0.05 were identified as DEGs using a bulk-RNAseq dataset for UC. The raw data was retrieved from GEO under accession ID: GSE57945 [22]. The genomic coordinates of DEGs were downloaded from the NCBI gene database according to the corresponding gene name (Gene ID). A total of 46,939 SNPs were extracted from our genotype data, of which 46,818 SNPs were present in meta-GWAS summary statistics.

SNPs associated with tissue-specific eQTLs. We obtained tissue-specific eQTLs data from the research conducted by Hu S et al. [23]. By analyzing the genotype data of 165 individuals diagnosed with IBD (UC = 68, Crohn’s disease  =  97) and the messenger RNA (mRNA)-sequencing data of 280 intestinal mucosal biopsy samples from these patients (112 samples from inflamed tissue and 168 from non-inflamed tissue), Hu S et al. studied the presence of eQTLs in IBD, identified 8881 intestinal eQTLs, and revealed 128 eQTLs that colocalized with UC. Comparing 8881 intestinal eQTLs and 128 UC eQTLs with our genotype data, we matched 8327 SNPs and 120 SNPs, respectively, and only 8250 and 117 of these variants were present in the meta-GWAS summary statistics.

In total, 7 PRS statistics were constructed for each sample using different SNP sets: (1) weighted PRSs using significant SNPs; (2) weighted PRSs using genome-wide SNPs; (3) weighted PRSs using SNPs in the DEGs; (4) weighted PRSs using intestinal eQTLs; (5) weighted PRSs using UC eQTLs; (6) unweighted PRSs using intestinal eQTLs; (7) unweighted PRSs using UC eQTLs.

We built unweighted PRSs and weighted PRSs by using ‘-score’ of PLINK (V1.9). The count of alleles associated with higher UC risk (0, 1, 2) was tallied and summed to calculate an unweighted PRS for each individual. A weighted PRS was calculated as the sum of risk alleles carried by each individual multiplied by their corresponding SNP weights, where the weights were defined as the log odds ratios (ORs) from the meta-GWAS summary statistics. The formulas for the unweighted and weighted PRSs, respectively, are ${PRS}_{j}=\frac{\sum_{i}^{N}G_{ij}}{P\times M_{j}}$, ${PRS}_{j}=\frac{\sum_{i}^{N}S_{i}\times G_{ij}}{P\times M_{j}}$, where *j* = jth subject, *N* = total number of SNPs, *S_i_* = the effect size of SNP *i*, *G_ij_* = number of effect alleles in sample *j* (0, 1, 2), *p* = the ploidy of the sample (is generally 2 for humans), and *M_j_* = the number of non-missing SNPs observed in sample *j* [24].

Those PRS models were assessed in R using logistic regression. To mitigate the potential problem of overfitting, a 5-fold cross-validation was performed 100 times for each model. The generation of the area under the receiver operating characteristic curve (AUC) for the covariates-only model (including age, sex, and the top 10 PCs) and the model incorporating the PRS along with covariates provided estimates of the models’ overall accuracy of case–control classification. We also evaluated each quintile PRS with UC in a logistic regression model and calculated the OR, with the lowest quintile serving as the reference group. Age, sex, and the top 10 PCs were included as covariates in the model.

### 2.10. Genetic Correlation Between Traits

Genetic correlation assesses the average genome-wide correlation in variants effects between phenotypes. The genetic correlation between UC and type 1 diabetes (T1D) was estimated using LDSC (https://github.com/bulik/ldsc, v1.0.1) and precalculated LD scores from the 1000 Genome European reference panel. The alleles in GWAS summary statistics were checked against HapMap3 SNPs used to evaluate the LD scores using “—merge-alleles” in the munge_sumstats.py function. Genetic correlation between UC and T1D was performed using the GWAS summary derived from cases and matched controls in the UK biobank.

## 3. Results

### 3.1. Variant-Level Association Analysis

**Figure 2:**
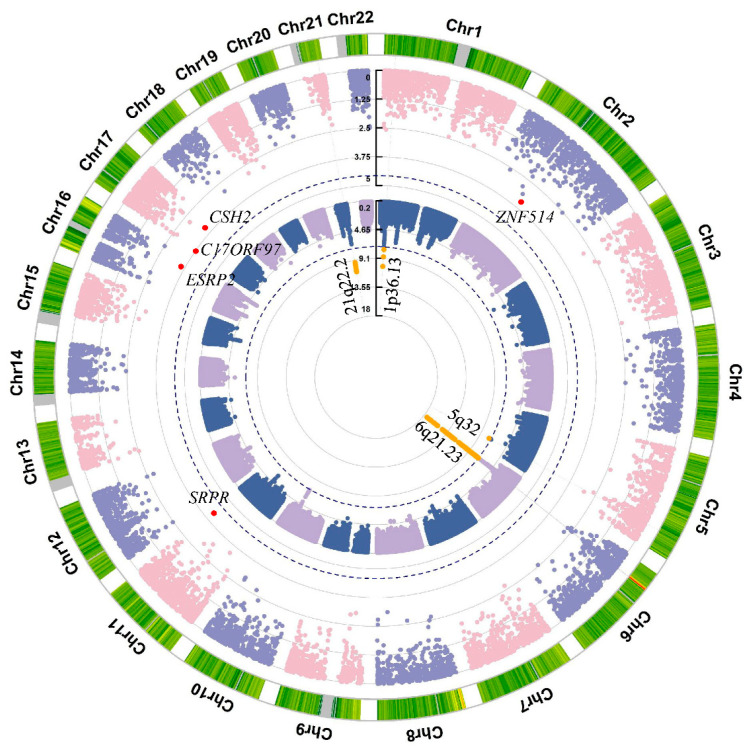
Genome-wide association analysis and variant collapsing analysis results for ulcerative colitis. The inner circle represents the circular Manhattan plot for the genome-wide association analysis results. The blue dashed line denotes the genome-wide significance level (*p* = 5 × 10^−8^). The locus reaching the genome-wide significance level is highlighted in orange. The outer circle represents the Manhattan plot for the variant collapsing analysis results. The blue dash line denotes the Bonferroni-adjusted significance level (*p* = 3.05 × 10^−6^). The top five significant genes resulting from variant collapsing analysis are labeled and highlighted in red in the plot. The outermost ring displays the density of mutation counts in each bin of chromosomes, with the color gradient from green to red indicating low to high density. The default parameter was used for the density displaying.

For the summary statistics at the variant level, a genomic inflation factor of 1.02 was obtained for all variants with an MAF > 0.01 (Figure S2), indicating the results were not likely to be inflated by implicit population structures. Overall, 608 variants reached the genome-wide significance (*p* = 5 × 10^−8^), with 592 of these variants being located in the human leukocyte antigen (HLA) region (Table S1). This finding was consistent with previous GWASs of UC in other populations, which have also identified the primary significant associations in the HLA region on chromosome 6, with a main peak being in the region of the HLA class II genes (Figure 2). The other significant variants were distributed on the loci of chromosome 1 (3 variants), chromosome 5 (1 variant), and chromosome 21 (12 variants). The variant-level associations were further clumped (see Methods), resulting in 25 clumps. Chromosome 6 included 21 index variants, most of which were in the locus containing HLA-DR (3 variants) and HLA-DQ (5 variants). The other index variants resided in clumps formed on chromosome 1 (two variants), chromosome 5 (one variant) and chromosome 21 (one variant). The main signal in the HLA region was rs9269070 (chr6:32440451:G:A, *p* = 3.56 × 10^−18^, OR = 1.325 [95% CI: 1.243–1.411]), which was an intron variant of HLA-DRB9. Other than the HLA region, two index variants were located in chromosome 1, rs4654925 (chr1:20227723:G:C, *p* = 1.85 × 10^−8^, OR = 0.835 [95% CI: 0.785–0.889]) residing in the intron of *OTUD3*, which has been one of the strongest associated loci reported previously in a UC study [25]. The other variant, rs2314757 (chr1:20131771:C:T, *p* = 4.82 × 10^−11^, OR = 0.81 [95% CI: 0.76–0.862]), was an intergenic variant. Only one significant variant was identified on chromosome 5, rs6869382 (chr5:145322071:C:T, *p* = 2.48 × 10^−8^, OR = 0.826 [95% CI: 0.772–0.883]), which is a novel site, residing in the non-coding region of *SH3RF2*. Chromosome 21 also had only one significant variant, rs2836882 (chr21:40466570:G:A, *p* = 1.23 × 10^−11^, OR = 0.778 [95% CI: 0.724–0.837]), which was a protective site located in an intergenic region, reported in a previous UC GWAS [26].

### 3.2. Gene-Level Association Analysis by Aggregating High Impact Rare Variants

**Figure 3:**
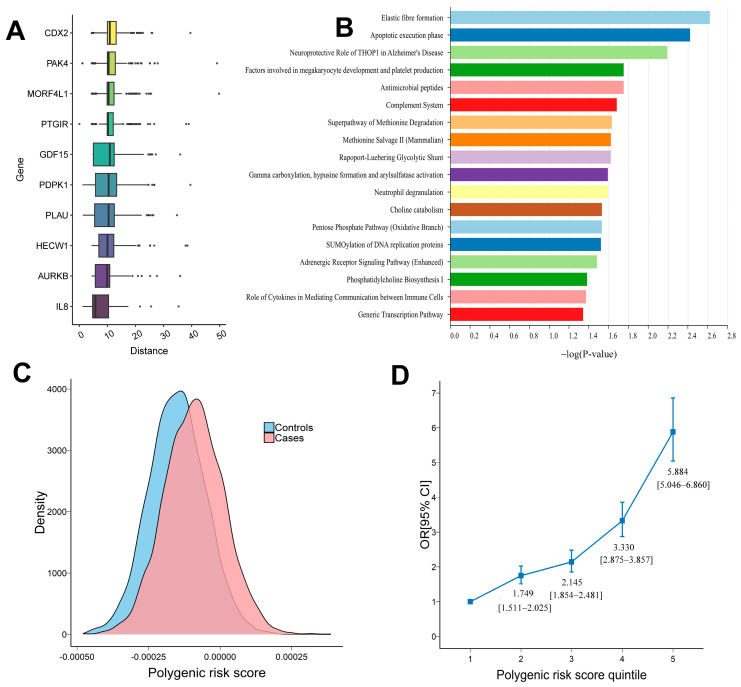
(**A**) The boxplot of biological distance to known UC-associated genes for candidate genes. The top ten genes are displayed in the plot. Shorter distance means biologically closer to UC genes. (**B**) IPA pathway analysis based on significant genes derived from high-impact rare variant collapsing analysis. Pathways with *p* < 0.05 are displayed. (**C**) Density of the most optimal polygenic risk scores of individuals in cases and controls. (**D**) Disease odds ratio (OR) for the second to fifth quintile of the most optimal polygenic risk score (the first quintile used as reference). Vertical bars demarcate 95% confidence intervals. The odds ratio is denoted as the odds of UC cases in quintiles over the odds of UC cases in the reference panel (the lowest quintile).

In the gene-based rare variants (MAF < 0.01) collapsing analysis, we did not observe any genes that reached the Bonferroni-adjusted significance (*p* = 3.05 × 10^−6^) among 16,379 genes containing available high-impact rare sites (Table S2). The three most significant genes identified in this study were *SRPR* (*p* = 7.34 × 10^−5^), *C17orf97* (*p* = 1.81 × 10^−4^), and *ESRP2* (*p* = 2.41 × 10^−4^) (Figure 2). *ESRP2*, as well as *ESRP1*, are known to play crucial roles in the maintenance of epithelial tight junction integrity [27]. The disruption of *ESRP2* may cause the breakdown of epithelial barrier integrity, which is a hallmark in the gut of IBD patients. However, it was not significantly overexpressed in UC cases according to the RNA-seq analysis results, where only *SRPR* reached the nominal significance for differential expression (log2FoldChange = 0.166, *p*-value = 0.018). We did not find strong evidence to support the associations of *SRPR* and *C17orf97*. We prioritized the candidate genes by assessing their biological distance to the UC-associated genes using the HGC [19]. *IL8* showed the shortest average proximity to the known UC genes (Figure 3A and Table S3). We then conducted IPA to evaluate whether there are biological functions that are implicated in the genes reaching nominal significance at an alpha level of 0.01. The results revealed 21 statistically significant pathways associated with the gene set, with elastic fiber formation being the most significant pathway (Figure 3B and Table S4).

### 3.3. Polygenic Risk Score Analysis

We evaluated the performance of eight different models in accurately classifying UC cases and controls by using the AUC metric. Table 1 shows the best results and Tables S5–S10 reports the results for each PRS model. Most PRS models provided predictive power above and beyond the covariates-only model. By comparing computational methods used to generate PRSs, it was observed that the weighted PRS exhibited superior overall performance for distinguishing between UC cases and controls, as it combined the effects of each SNP to the occurrence of the UC, compared to the unweighted PRS.

We subsequently screened some SNPs in the genes that are potentially involved in the etiology of the UC, and some SNPs with important biological effects, to generate a PRS. Although selecting SNPs in the DEGs helped us narrow the candidate SNPs for the PRS, it failed to improve the performance of the PRS. Instead, it performed worse than the PRS based on genome-wide SNPs at each r^2^ and *p*-value threshold (Tables S5 and S6). The weighted PRS derived from these eQTLs demonstrated a modest classification potential with an AUC of 0.589 for UC eQTLs and 0.602 for intestinal eQTLs. Although the AUCs of those two PRSs were not as high as the AUC of the best weighted PRS based on genome-wide SNPs, they were comparable to the AUC values of the weighted PRS based on significant SNPs identified from the meta-GWAS. When calculating unweighted PRSs using UC eQTLs and intestinal eQTLs, we also made a selection of representative SNPs that were identified according to LD pruning using the “-indep-pairwise” with a window size of 250 kb, a step size of 50, and a r^2^ of 0.05, 0.1, 0.2, 0.4, 0.6, or 0.8. Among all unweighted PRSs, their performance was poor, with the maximum AUC being only 0.527.

In the best-performing PRS, which was a weighted PRS using genome-wide SNPs (r^2^ = 0.8, *p* = 5 × 10^−2^), a higher PRS was significantly associated with an increased risk of UC (Figure 3C). Individuals in the highest quintile of the PRS had a 5.884-fold increased risk of UC compared with the bottom 20% of individuals. A shape plot was generated to illustrate the differences in risk of the PRS quintile from the first quintile, as depicted in Figure 3D.

### 3.4. Variant-Level and PRS-Based Phenome-Wide Association Analysis

**Figure 4:**
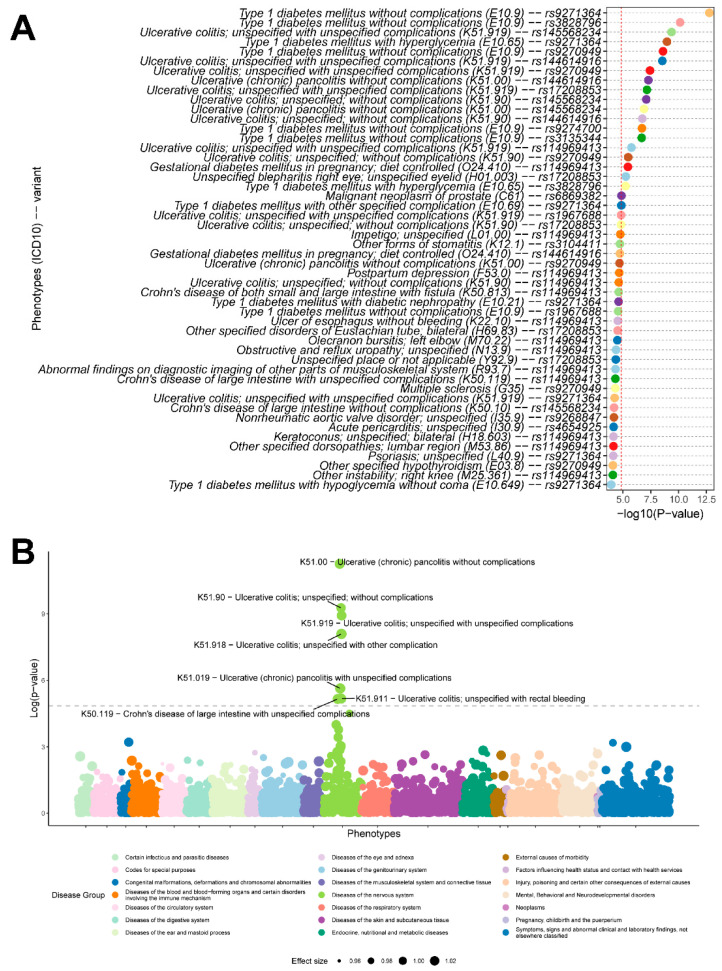
Phenome-wide association analysis results based on ICD-10 codes. (**A**) Results of phenome-wide association analysis for genome-wide significant variants using ICD-10 codes. The red dashed line indicates the Bonferroni-adjusted *p*-value threshold. The labels on *y*-axis denote the associations between diagnosis phenotypes and the variants. The top 50 associations are displayed. (**B**) The Manhattan plot for PRS-based phenome-wide association study using ICD-10 codes. The dashed line denotes the Bonferroni-adjusted significance threshold. Disease groups were generated according to the main categories of ICD-10.

At the variant level, 25 index SNPs in clumps of variants reached the genome-wide significance, in which 19 variants were available in the BioMe BioBank imputation dataset. These 19 variants were further evaluated by conducting a variant-level PheWAS. As a result, 21 associations passed the phenome-wide significance, in which the association with UC was identified (K51.919, K51.00, K51.90). The variant rs145568234 (chr6:32247045:T:G) was strongly associated with the risk of UC, with an OR of 15.27 (*p* = 4.36 × 10^−10^). Type 1 diabetes (T1D) was the most relevant condition among the significant associations, with five variants: The rs9271364 [chr6:32586787:A:G] was protective for E10.9 (OR = 0.61, *p* = 1.70 × 10^−13^), E10.65 (OR = 0.51, *p* = 1.11 × 10^−9^), and E10.69 (OR = 0.36, *p* = 1.40 × 10^−5^). The rs3828796 [chr6:32635974:A:G] increased the risk of the E10.9 (OR = 1.61, *p* = 7.35 × 10^−11^) and E10.65 (OR = 1.70, *p* = 5.88 × 10^−6^). The rs9270949 [chr6:32572975:T:C] was protective for E10.9 (OR = 0.59, *p* = 2.52 × 10^−9^). The rs9274700 [chr6:32637180:A:G] increased the risk of the E10.9 (OR = 1.41, *p* = 1.98 × 10^−11^). The rs3135344 [chr6:32395036:C:T] was protective for E10.9 (OR = 0.63, *p* = 2.09 × 10^−7^). Among the significant results reaching the phenome-wide significance, ten signals were associated with UC, whereas eight associations were related to T1D. The remaining conditions included “Gestational diabetes mellitus in pregnancy (ICD10: O24.410)”, “Malignant neoplasm of prostate (ICD10: C61)”, and “Unspecified blepharitis right eye (ICD10: H01.003)” [28,29,30]. Almost all variants involved in the associations that reached phenome-wide significance level were from the HLA region, except for rs6869382 [chr5:145322071:C:T], which was protective (OR = 0.73, *p* = 1.37 × 10^−5^) for malignant neoplasm of the prostate (Figure 4A and Table S11). The other novel variant, rs2314757, which is protective for UC, is also associated with reduced risk of “other specified cardiac arrhythmias” (ICD-10: I49.8), a phenotype of clinical relevance given the established link between IBD and arrhythmias, thereby implicating a potential pleiotropic genetic factor (Table S11).

The PRS was generated for 53,449 individuals who passed the QC procedure in BioMe BioBank, based on the 164,107 variants used in the best UC PRS model in the UK Biobank. The BioMe imputation data covered 156,962 (95.64%) of the 164,107 variants, which were used for calculating PRS for samples. After executing a PRS-based PheWAS on the filtered phenotypes as described above, the most significant phenotype associated with PRS was ulcerative (chronic) pancolitis (K51.00, *p* = 5.42 × 10^−12^, OR = 1.03). The second most significant association was with ulcerative colitis (K51.90, *p* = 5.36 × 10^−10^, OR = 1.02) (Figure 4B). There were four more granular ICD10 codes related to UC reaching the Bonferroni-adjusted threshold (*p* = 1.43 × 10^−5^), including K51.919 (*p* = 1.19 × 10^−9^, OR = 1.02), K51.918 (*p* = 8.06 × 10^−9^, OR = 1.03), K51.019 (*p* = 2.33 × 10^−6^, OR = 1.03), and K51.911 (*p* = 6.75 × 10^−6^, OR = 1.03). The other phenotype that passed the phenome-wide significance level was Crohn’s disease (K50.119, *p* = 7.02 × 10^−6^, OR = 1.02) (Table S12).

**Figure 5:**
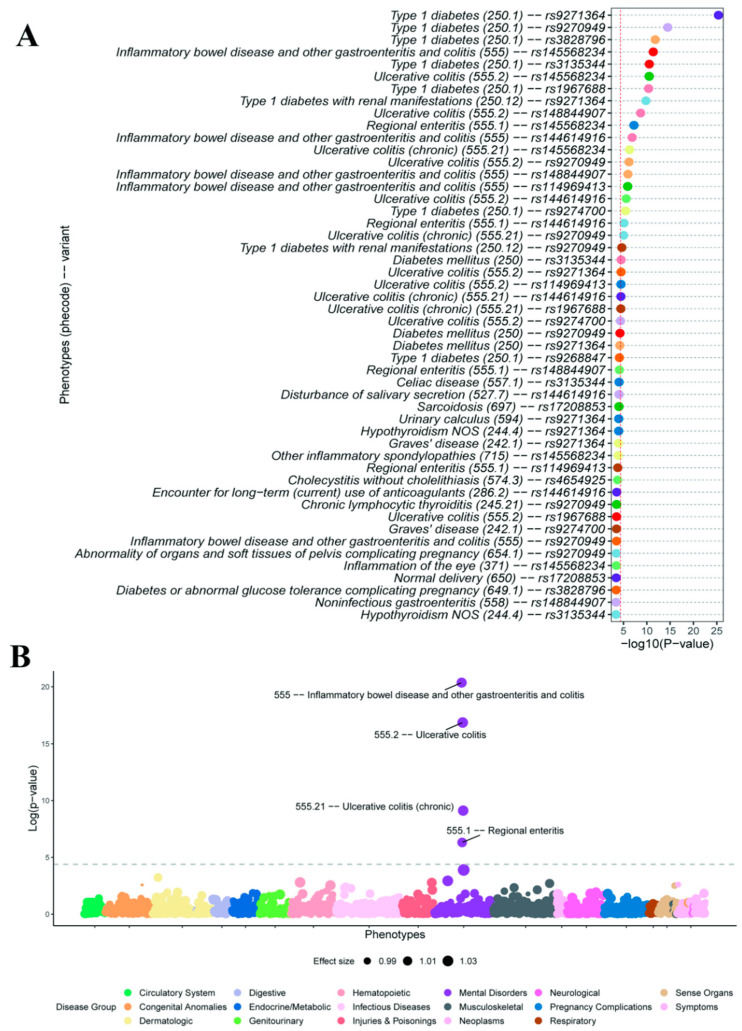
Variant-level and PRS-based phenome-wide association analysis results based on phecodes. (**A**) Results of phenome-wide association analysis for genome-wide significant variants using phecodes. The red dashed line indicates the Bonferroni-adjusted *p*-value threshold. The labels on *y*-axis denote the associations between diagnosis phenotypes (phecodes) and the variants. The top 50 associations are displayed in the figure. (**B**) The Manhattan plot for PRS-based phenome-wide association study using phecodes. The dashed line denotes the Bonferroni-adjusted significance threshold. Disease groups are generated according to the categories of phecodes.

In order to increase the power to identify clinical phenotypes associated with UC, we additionally performed variant- and PRS-based PheWAS using phecodes (see Methods). In the variant-level PheWAS, 20 associations reached the phenome-wide significance, in which 10 associations were IBD- or UC-related (Figure 5A). Type 1 diabetes (phecode 250.1) had the most significant association with a UC variant (rs9271364, *p* = 4.46 × 10^−26^, OR = 0.51). In total, T1D occupied eight positions among associations that surpassed the phenome-wide significance. The remaining significant associations included “Regional enteritis (phecode 555.1)”, which had two significant hits (Table S13). Regional enteritis (Crohn’s disease) is another major form of IBD, affecting the ileum and colon [31].

In the PRS-based PheWAS results, four phenotypes surpassing the phenome-wide significance level (Figure 5B), “Inflammatory bowel disease and other gastroenteritis and colitis” (phecode 555), “Ulcerative colitis” (phecode 555.2), “Ulcerative colitis (chronic)” (phecode 555.21), and “Regional enteritis” (phecode 555.1). Among the non-IBD phenotypes, “Hypothyroidism NOS” (phecode 244.4) was the most significant association with the UC PRS (*p* = 6.03 × 10^−4^) (Table S14).

## 4. Discussion

Our variant-level association results were largely consistent with previous studies. The HLA region contained the majority of risk variants reaching genome-wide significance, with the strongest signals located in the *HLA-DR* and *HLA-DQ* genes. In our dataset, the most significant variant was rs9269070 (*p* = 3.56 × 10^−18^, OR = 1.32 [1.24–1.41], chr6:32472674:G:A in GRCh38; chr6:32440451:G:A in GRCh37), which was also identified as an index variant by PLINK. However, there is no prior evidence supporting rs9269070 as a causal variant for UC. It is possible that this variant is in linkage disequilibrium (LD) with another causal variant in the region. Another locus previously reported to be strongly associated with UC is *OTUD3*, where a UC risk variant (rs773646156) was described in a recent study. However, this variant was not included in our analysis due to its absence from the imputed UK Biobank dataset [32]. Instead, we identified a protective intronic variant within *OTUD3*. *OTUD3* has been recognized as a critical protective gene in UC, as it helps maintain gut homeostasis by finely tuning inflammatory responses in fibroblasts. Risk mutations in *OTUD3* can synergize with dysbiotic microbiome-derived cGAMP to promote disease progression [33]. In addition, we detected twelve variants at 21q22.2 that were significantly associated with UC. The leading variant, rs2836882, was protective (OR = 0.77 [0.72–0.84], *p* = 1.23 × 10^−11^). A previous study also reported a protective effect of rs2836878 at 21q22 (OR = 0.73) [34], consistent with our findings for rs2836882 (OR = 0.78 [0.72–0.83], *p* = 2.07 × 10^−11^). These variants are located in the intergenic region between *TNFRSF6B* and *PSMG1*, both of which have been implicated in IBD pathogenesis [35,36,37]. Notably, the 21q22 region harbors loci associated with pediatric-onset IBD, warranting further investigation to disentangle the signals within this region.

In the variant collapsing analysis, 155 genes reached nominal significance at *p* = 0.01. Pathway analysis of these genes identified 21 pathways with *p* < 0.05, of which elastic fiber formation was the most significant. Interestingly, loss of elastic fibers has been reported in long-standing UC [38], suggesting a potential link between impaired elastic fiber formation and the high-impact rare variants identified in this study. Among the significant genes, *ESRP2*, which is implicated in intestinal barrier integrity, represents a potential therapeutic target [39,40]. To further prioritize candidate genes, we applied human gene connectome methods, which highlighted *IL8* as the top gene due to its biological proximity to known UC-associated genes. *IL8* is specifically expressed in inflamed mucosa and plays a critical role in UC progression [41]. Although our results suggest that high-impact rare variants contribute to UC susceptibility, larger sample sizes and additional analyses are needed to validate these findings and pinpoint causal variants.

Using genetically identified European UC cases and controls, we evaluated the performance of a PRS-derived from large-scale European GWAS summary statistics in identifying high-risk individuals. The predictive utility of PRS has been demonstrated across multiple diseases, making it a promising tool for early detection and preventive interventions [42,43,44,45]. In our analysis, incorporating PRS significantly improved risk stratification compared with a covariate-only model (age, sex, and the top 10 PCs), increasing the AUC by up to 0.153. Despite the modest AUC of 0.66 for the best-performing PRS, which currently limits its direct clinical utility, individuals in the highest quintile of the best-performing PRS exhibited a 5.88-fold increased risk of UC, indicating potential clinical relevance. These findings suggest that PRS may serve as a foundation for future individualized risk prediction. Meanwhile, the performance of our polygenic risk score is comparable to that reported in previous studies for UC [46]. The generally modest predictive accuracy of genetics-based models in UC is likely attributable to the high genetic heterogeneity of the disease.

We further evaluated factors that may influence PRS performance by filtering SNP sets under different significance thresholds and functional annotations. The PRS derived from genome-wide significant SNPs in the latest and largest UC meta-GWAS demonstrated limited predictive power, with an AUC of 0.590. In contrast, extending SNP inclusion to the genome-wide nominally significant variants substantially improved predictive accuracy [47]. The most optimal PRS in this study was derived using the “clumping + thresholding” approach, which incorporated genome-wide SNPs. Rapidly screening SNPs based on their GWAS *p*-values enables the retention of important predictive variants, particularly for diseases like UC with incompletely characterized genetic components. Adjusting the LD *r^2^* and *p*-value thresholds allows for the inclusion of both known UC-associated variants and potential risk loci not detected in previous GWASs. However, caution is warranted, as overly relaxed thresholds can introduce large numbers of SNPs with weak or no phenotypic relevance. The inclusion of correlated SNPs without independent signals or with limited information may generate noise and ultimately impair PRS performance [24,45].

Determining which SNPs to include in a PRS is a critical step for its application in disease risk assessment. However, there is still no consensus on the optimal methods for PRS calculation or the criteria for SNP selection [24,48]. Consequently, exploring different SNP selection strategies is essential. Based on LD and *p*-value thresholds, we conducted additional SNP screening using differentially expressed genes (DEGs) or eQTL SNPs to generate PRSs and assess whether their predictive performance could be improved. The weighted PRSs incorporating SNPs from DEGs yielded AUCs ranging from 0.513 to 0.532, insufficient for meaningful risk classification. This limited performance likely stems from the fact that most SNPs within DEGs are not causally linked to the observed differential gene expression and have weak associations with UC.

EQTLs, genetic loci that influence gene expression [49] were similarly evaluated for PRS construction. However, using eQTLs did not enhance PRS performance as anticipated. This may be because the eQTLs were derived from inflamed and non-inflamed intestinal mucosal biopsies from IBD patients, and thus may not substantially impact UC development [23]. These findings underscore the importance of sample and tissue selection when studying DEGs and eQTLs, which could improve PRS performance if more relevant data were available. Overall, whether filtering SNPs by significance or by functional annotations, the fundamental nature of PRS construction, aggregating SNP effects within individuals, remains unchanged. Adjusting filtering criteria primarily alters the number of included SNPs without truly incorporating functional effects. Therefore, enhancing PRS performance by integrating SNP functionality requires more sophisticated approaches.

Since UC is an autoimmune disease with a higher likelihood of multimorbidity compared to Crohn’s disease, and PRS can help identify traits sharing genetic liability with UC [9], we performed a PRS-based PheWAS to detect comorbidities in addition to a variant-level PheWAS using genome-wide significant variants. The variant-level PheWAS implemented in the BioMe Biobank replicated two UC associations identified in the original GWAS from the UK Biobank. Initially, samples were divided into subgroups based on original ICD-10 codes, allowing for analysis of smaller, more granular disease categories. Several associations between these disease subgroups and UC variants were identified. Type 1 diabetes (T1D) emerged as the most significant phenotype, with eight hits reaching phenome-wide significance, including five variants from the HLA region. Among these, rs9270949 was associated with both UC and T1D, but exerted effects in opposite directions. This phenomenon, previously reported [50], wherein variants exhibit opposite effects across T1D, Crohn’s disease, and UC, may explain the increased risk of diabetes in Crohn’s disease patients but not in UC [51]. Our PheWAS and genetic correlation analyses indicate that UC and T1D share genetic liability, supporting recent studies reporting bidirectional associations between IBD and T1D [52]. Other significant associations included gestational diabetes mellitus, linked to UC via the shared risk variant rs114969413. This finding aligns with reports that pregnant women with IBD have higher odds of developing gestational diabetes [53]. Other notable associations included unspecified blepharitis of the right eye, likely representing an ophthalmic manifestation in IBD patients [29], and malignant neoplasm of the prostate. A recent study reported a link between IBD, particularly UC, and prostate cancer [28]. Consistently, rs6869382, which was associated with reduced UC risk in the UK Biobank, also conferred protection against prostate cancer in our variant-level PheWAS.

Our PRS-based PheWAS revealed several clinical associations strongly linked with UC. The cumulative effect of nominally significant variants in GWAS was highly associated with UC (K51.00, 327 cases, OR = 1.03, *p* = 5.42 × 10^−12^). Although UC samples were assigned to different UC sub-phenotypes based on ICD-10 codes, which may have reduced the power to detect associations, all signals that survived Bonferroni correction were IBD-related diagnoses. Following the most significant association, the 2nd to 7th associations were also related to UC. The final significant association, just surpassing the phenome-wide threshold, was Crohn’s disease of the large intestine (K50.119). This was followed by three Crohn’s disease phenotypes that nearly reached significance, including Crohn’s disease of the small intestine (K50.00), Crohn’s disease (K50.90), and Crohn’s disease of both the small and large intestine with fistula (K50.813). The association of Crohn’s disease with UC PRS likely reflects the shared genetic architecture between UC and Crohn’s disease, resulting in overlapping genetic signals [54]. The observed genetic overlap between UC and T1D is consistent with the clinical presentation of autoimmune polyglandular syndrome, wherein patients develop multiple autoimmune disorders. This shared genetic architecture may partly explain the clinical co-occurrence of these conditions. Furthermore, from a clinical management perspective, it is important to consider that standard treatments for UC, such as systemic glucocorticoids, are known to elevate blood glucose levels and may unmask or exacerbate diabetes, adding complexity to the care of patients affected by both diseases. The UC PRS, which represents the cumulative effect of UC-associated variants, improved the specificity for identifying IBD-related conditions. This may explain why T1D reached a nominal significance level (*p* = 7.33 × 10^−3^) in the variant-level PheWAS, but its association was not as strong as that of IBD. While a single variant can be associated with multiple conditions due to pleiotropic effects or shared biological processes, the accumulated effect of multiple risk variants can drive specific disease traits. This pattern could be utilized to develop a new approach for identifying comorbidities: by deliberately reducing the specificity for single-disease PRS traits, one could increase the sensitivity for detecting multi-morbidity patterns.

To enhance statistical power and clinical interpretability, we also performed variant-level and PRS-based phenome-wide association studies (PheWAS) using phecodes, which aggregate related ICD-10 codes into clinically meaningful phenotypes. The phecode-based results were largely consistent with those derived from original ICD-10 codes, though the former yielded a greater number of significant associations. At the variant level, PheWAS identified UC and T1D as the two primary groups of associations. In contrast, both ICD10-based and phecode-based PRS-PheWASs consistently identified UC as the most significant association with the UC PRS. The variant-level PheWAS demonstrated higher sensitivity in detecting comorbidities, irrespective of the coding system used, whereas the PRS-based PheWAS showed greater specificity by predominantly identifying phenotypes directly related to UC. Thus, rational integration of variant-level and PRS-based PheWASs may provide a powerful strategy for uncovering disease-related comorbidities while minimizing ambiguous signals. Future methodological refinements will be required to define the optimal balance between sensitivity and specificity when applying these approaches to large-scale, diverse biobank populations.

## 5. Conclusions

In summary, this study delineated both novel and established genetic loci associated with ulcerative colitis and demonstrated the utility of PRS for disease risk stratification. Both variant-level and PRS-based PheWASs highlighted clinically relevant comorbidities, most notably type 1 diabetes, underscoring a shared genetic liability between UC and other autoimmune diseases. These findings enhance our understanding of UC pathogenesis, support the role of PRS in predicting UC-related traits, and provide a foundation for future work on early risk stratification and comorbidity management in large-scale biobank settings.

## Figures and Tables

**Table 1:** Performance evaluation of covariates-only model and different PRS models.

Models		AUC	Number of SNPs
Covariates-only		0.513	
Weighted PRS	Genome-wide significant SNPs	0.590	47
	Genome-wide SNPs (r^2^ = 0.8, *p* = 5 × 10^−2^)	0.665	164,107
	Selected DEG-SNPs (r^2^ = 0.6, *p* = 5 × 10^−4^)	0.532	73
	Selected intestinal eQTLs (r^2^ = 0.6, *p* = 1)	0.602	7822
	Selected UC eQTLs (r^2^ = 0.1, *p* = 1)	0.589	89
Unweighted PRS	Selected intestinal eQTLs (all sites)	0.513	8327
	Selected UC eQTLs (r^2^ = 0.05)	0.527	74

## Data Availability

This study was conducted using the UK Biobank Resource under Application 53074. The summary statistics of IBD derived from a latest meta-analysis GWAS based on East Asian and Europeans is available on the IIBDGC website (https://www.ibdgenetics.org, accessed on *5* May 2025).

## Supplementary Materials

The following supporting information can be downloaded at https://www.mdpi.com/article/10.3390/genes16121431/s1. Table S1. GWAS results of significant SNPs associated with ulcerative colitis (P<5e-8) (Provided in separated file). Table S2 SKAT-O analysis on ulcerative colitis using high impact rare variants (Provided in separated file). Table S3. Gene prioritization results for variant collapsing analysis using Human Gene Connectome. Table S4. IPA pathway analysis based on significant genes derived from high impact rare variants collapsing analysis (Provided in separated file). Table S5. Prediction accuracy of the weighted PRSs built with genome-wide SNPs (Provided in separated file). Table S6 Prediction accuracy of the weighted PRSs built with SNPs in the differential expression genes (Provided in separated file). Table S7. Prediction accuracy of the weighted PRSs built with intestinal eQTLs (Provided in separated file). Table S8. Prediction accuracy of the weighted PRSs built with UC eQTLs (Provided in separated file). Table S9. Prediction accuracy of the unweighted PRSs built with intestinal eQTLs (Provided in separated file). Table S10. Prediction accuracy of the unweighted PRSs built with UC eQTLs. (Provided in separated file). Table S11. Variant-level PheWAS analysis results based on ICD10 codes using Mount Sinai BioMe Biobank. (Provided in separated file). Table S12. PRS-based PheWAS analysis results based on ICD10 codes using Mount Sinai BioMe Biobank imputation data. (Provided in separated file). Table S13. Variant-level PheWAS analysis results based on phecodes using Mount Sinai BioMe Biobank. (Provided in separated file). Table S14. PRS-based PheWAS analysis results based on phecodes using Mount Sinai BioMe Biobank. Figure S1. A principal component analysis plot for displaying genetically identified European samples for this study. Figure S2. The QQ plot of genome-wide association analysis for ulcerative colitis.

## Abbreviations

The following abbreviations are used in this manuscript:

UC	Ulcerative colitis
IBD	Inflammatory bowel disease
QC	Quality control
PCA	Principal component analysis
GWAS	Genome-wide association study
PheWAS	Phenome-wide association study
HGC	Human gene connectome
IPA	Ingenuity Pathways Analysis
PRS	Polygenic risk score
SNP	Single nucleotide polymorphism
eQTL	Expression quantitative trait locus
DEG	Differentially expressed gene
LD	Linkage disequilibrium
EHR	Electronic health records
HLA	Human leukocyte antigen
OR	Odds ratio
AUC	Area under the receiver operating characteristic curve
