# Supplementary material for: Unveiling Novel Traits Associated with Ulcerative Colitis via Phenome-Wide Associations Enhanced by Polygenic Risk Statistics
Source: Genes (Basel). 2025 Nov 30;16(12):1431. doi: 10.3390/genes16121431 (PMC12732458; doi:10.3390/genes16121431)

Unveiling novel traits associated with ulcerative colitis via phenome-wide associations enhanced by polygenic risk statistics

Supplementary files

Tables

Table S1. GWAS results of significant SNPs associated with ulcerative colitis (P<5e-8) (Provided in separated file)

Chr, Chromosome code. SNP, the variant identifier of SNP. BP-GrCh37, the base position of SNP based on the NCBI Genome Reference Consortium Human Build 37. OR, odds ratio. L95, U95, 95% confidence intervals of OR. SE, standard error. P, P value of GWAS.

Table S2 SKAT-O analysis on ulcerative colitis using high impact rare variants (Provided in separated file)

SetID, gene IDs tested by SKAT-O analyses. P.value, P values of SKAT. N.Marker.All, a number of SNPs in the genotype matrix. N.Marker.Test, a number of SNPs used for the test. MAC, the total minor allele count. M, the number of individuals with minor alleles. Method.bin, type of method to compute a P value.

Table S3. Gene prioritization results for variant collapsing analysis using Human Gene Connectome

Table S4. IPA pathway analysis based on significant genes derived from high impact rare variants collapsing analysis (Provided in separated file)

Table S5. Prediction accuracy of the weighted PRSs built with genome-wide SNPs (Provided in separated file)

r2, the linkage disequilibrium r2 value used to clump SNPs, P-value, the *P*-value threshold used to select SNPs. Number of SNPs, the number of SNPs used to calculate polygenic risk score.

Table S6 Prediction accuracy of the weighted PRSs built with SNPs in the differential expression genes (Provided in separated file)

r2, the linkage disequilibrium r2 value used to clump SNPs, P-value, the *P*-value threshold used to select SNPs. Number of SNPs, the number of SNPs used to calculate polygenic risk score.

Table S7. Prediction accuracy of the weighted PRSs built with intestinal eQTLs (Provided in separated file)

r2, the linkage disequilibrium r2 value used to clump SNPs, P-value, the *P*-value threshold used to select SNPs. Number of SNPs, the number of SNPs used to calculate polygenic risk score.

Table S8. Prediction accuracy of the weighted PRSs built with UC eQTLs (Provided in

separated file)

r2, the linkage disequilibrium r2 value used to clump SNPs, P-value, the *P*-value threshold used to select SNPs. Number of SNPs, the number of SNPs used to calculate polygenic risk score.

Table S9. Prediction accuracy of the unweighted PRSs built with intestinal eQTLs (Provided in separated file)

r2, the linkage disequilibrium r2 value used to clump SNPs, P-value, the *P*-value threshold used to select SNPs. Number of SNPs, the number of SNPs used to calculate polygenic risk score.

Table S10. Prediction accuracy of the unweighted PRSs built with UC eQTLs. (Provided in separated file)

r2, the linkage disequilibrium r2 value used to clump SNPs, P-value, the *P*-value threshold used to select SNPs. Number of SNPs, the number of SNPs used to calculate polygenic risk score.

Table S11. Variant-level PheWAS analysis results based on ICD10 codes using Mount Sinai BioMe Biobank. (Provided in separated file)

SNP, the predictor under study. Beta, the beta effect in logistic regression. SE, The standard error for the beta coefficient. N_total, number of cases and controls. HWE_p, p value in Hardy-Weinberg equilibrium exact test. N_no_snp, the number of records

with a missing predictor. Type, the type of regression model used.

Table S12. PRS-based PheWAS analysis results based on ICD10 codes using Mount Sinai BioMe Biobank imputation data. (Provided in separated file)

Estimate, the beta effects for the predictor in logistic regression. OR, odds ratio for the predictor. N_cases, number of cases for the phenotype. N_controls, number of controls used for the logistic regression analysis.

Table S13. Variant-level PheWAS analysis results based on phecodes using Mount Sinai BioMe Biobank. (Provided in separated file)

Beta, the beta effect in logistic regression. SE, The standard error for the beta coefficient. N_total, number of cases and controls. HWE_p, p value in Hardy-Weinberg equilibrium exact test. N_no_snp, the number of records with a missing predictor. Type, the type of regression model used. Combined_info, the phenotype, related phecode and associated variant rsID used for plot.

Table S14. PRS-based PheWAS analysis results based on phecodes using Mount Sinai BioMe Biobank. (Provided in separated file)

Description, the disease name of the representative phecode. Estimate, the beta effects for the predictor in logistic regression. N_cases, number of cases for the phenotype. N_controls, number of controls used for the logistic regression analysis. OR, odds ratio converted from beta effects.

Figures

Figure S1. A principal component analysis plot for displaying genetically identified European samples for this study.

Samples were compared to the reference panel including samples of European, East Asian, African (Yoruba in Ibadan) from the 1000 Genomes Phase 3 database. The genetically identified Europeans among the UK Biobank samples included in the study were highlighted in blue dots.

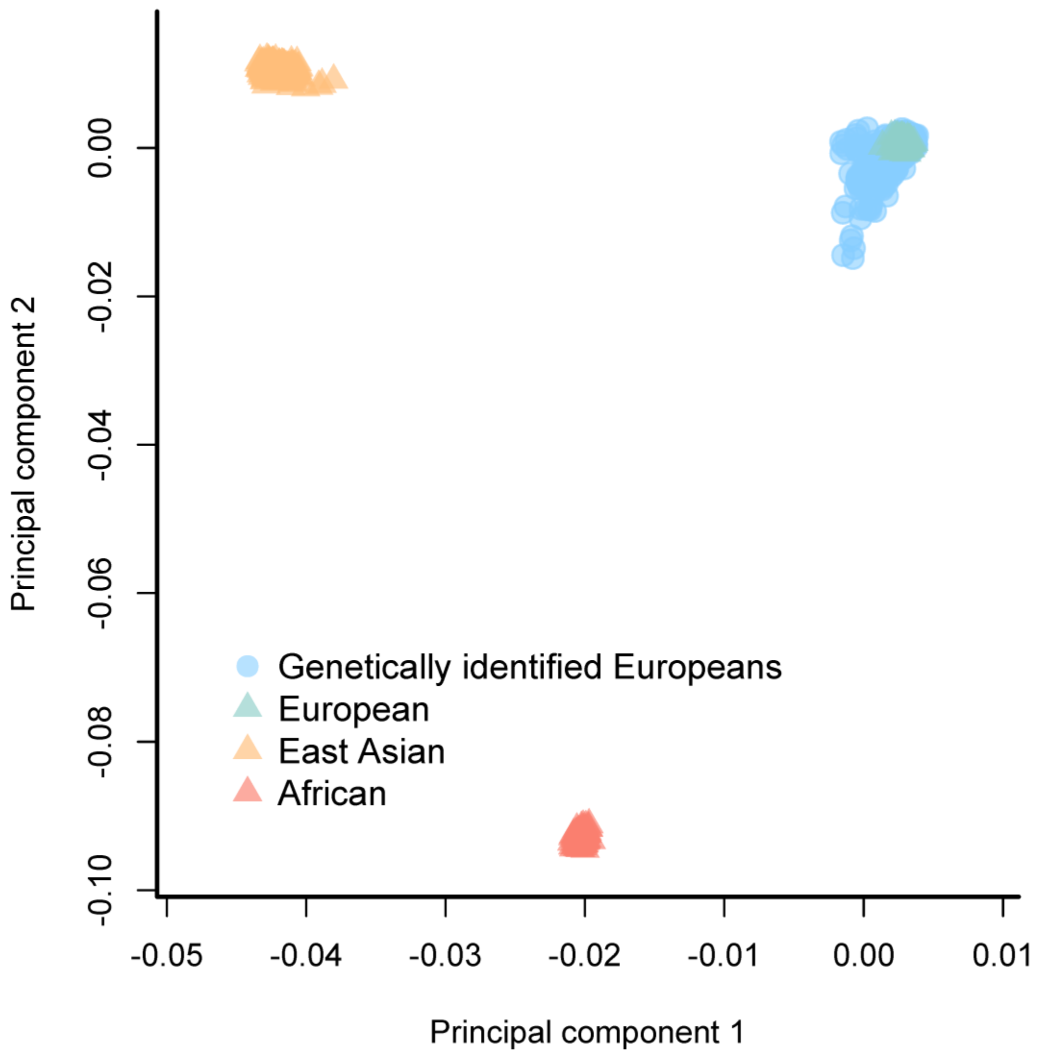

Figure S2. The QQ plot of genome-wide association analysis for ulcerative colitis.

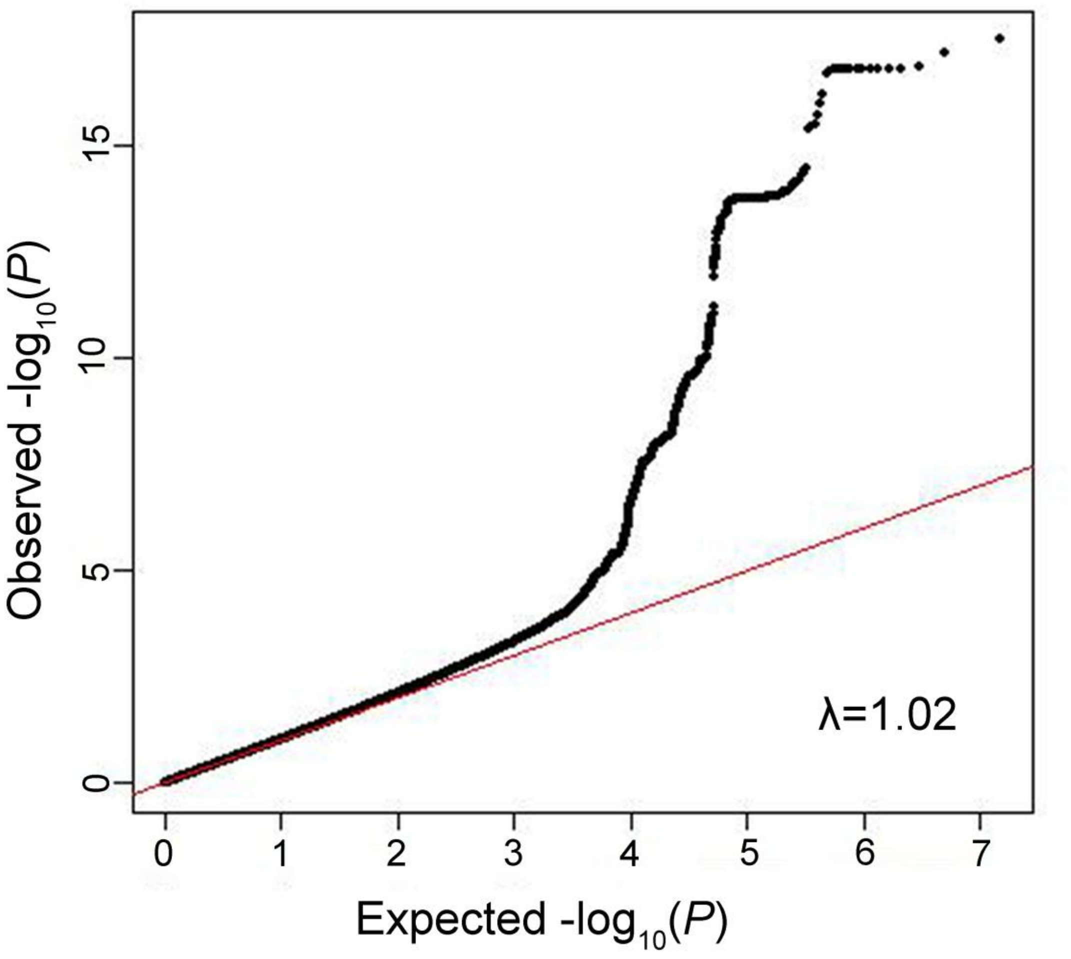

Supplement: Supplementary file 1 [file genes-16-01431-s001.zip › genes-3988001-supplementary.pdf]
